# Post-Integration Silencing of *piggyBac* Transposable Elements in *Aedes aegypti*

**DOI:** 10.1371/journal.pone.0068454

**Published:** 2013-07-04

**Authors:** Azhahianambi Palavesam, Caroline Esnault, David A. O’Brochta

**Affiliations:** 1 Department of Entomology, The Institute for Bioscience and Biotechnology Research, University of Maryland, College Park, Maryland, United States of America; 2 Institute for Bioscience and Biotechnology Research, Rockville, Maryland, United States of America; New Mexico State University, United States of America

## Abstract

The *piggyBac* transposon, originating in the genome of the Lepidoptera *Trichoplusia ni*, has a broad host range, making it useful for the development of a number of transposon-based functional genomic technologies including gene vectors, enhancer-, gene- and protein-traps. While capable of being used as a vector for the creation of transgenic insects and insect cell lines, *piggyBac* has very limited mobility once integrated into the genome of the yellow fever mosquito, *Aedes aegypti*. A transgenic *Aedes aegypti* cell line (AagPB8) was created containing three integrated *piggyBac* elements and the remobilization potential of the elements was tested. The integrated *piggyBac* elements in AagPB8 were transpositionally silent in the presence of functional transposase, which was shown to be capable of catalyzing the movement of plasmid-borne *piggyBac* elements in the same cells. The structural integrity of one of the integrated elements along with the quality of element-flanking DNA, which is known to influence transposition rates, were tested in *D. melanogaster*. The element was found to be structurally intact, capable of transposition and excision in the soma and germ-line of *Drosophila melanogaster,* and in a DNA sequence context highly conducive to element movement in *Drosophila melanogaster*. These data show that transpositional silencing of integrated *piggyBac* elements in the genome of *Aedes aegypti* appears to be a function of higher scale genome organization or perhaps epigenetic factors, and not due to structural defects or suboptimal integration sites.

## Introduction

DNA transposons are abundant and influential residents of many genomes and have been harnessed as gene vectors and functional genomics tools because their high mobility and simple structures permit them to be easily modified and manipulated [1], [2]. The long evolutionary history of DNA transposons in eukaryotic genomes has lead to the existence of numerous means by which the elements and their hosts’ genomes modulate and regulate their activity [3], [4], [5]. Understanding the various modes of DNA transposon regulation is important for understanding the dynamics and evolution of these elements within genomes and for being able to fully exploit the potential of these elements to be used as functional genomics tools.

The *piggyBac* element is a DNA transposon isolated originally from the genome of the moth *Trichoplusia ni* but has since been found in a range of other insect and non-insect species [6], [7], [8], [9], [10], [11], [12]. Like a number of other eukaryotic DNA transposons, *piggyBac* can transpose under some conditions in a host-independent manner making it an excellent platform upon which to build gene vectors and other functional genomics tools [13], [14], [15], [16], [17]. *piggyBac* has been used as a gene vector for the creation of growing number of transgenic insects of a wide range of species and in a few of those species more sophisticated *piggyBac*-based forward genetics and functional genomics tools have also been successfully developed [18]. For example, in *Drosophila melanogaster*, *Tribolium castaneum, Bombyx mori* and *Ceratitis capitata, piggyBac* has been an effective tool for transposon mutagenesis and enhancer/promoter trapping because it can be readily remobilized once it has integrated into the genome [16], [19], [20], [21], [22], [23]. Recently, it has been shown to have the same potential in the mosquito *Anopheles stephensi* [24]. In the mosquito *Aedes aegypti* the post-integration behavior of *piggyBac* is somewhat different in that integrated elements cannot be remobilized [25]. Sethuraman et al. (2007) attempted to remobilize five *piggyBac* elements located in different regions of the genome by providing *piggyBac* transposase from integrated *piggyBac*-expressing transgenes. Somatic and germ-line mobility of *piggyBac* were undetectable [25]. This post-integration silencing of *piggyBac* in *Ae. aegypti* is significant because it severely restricts the application of *piggyBac*-based genomics tools in this important vector of human pathogens. In addition, it could also reflect the existence of a systemic system for responding to the presence of new transposons within the genome of *Ae. aegypti* that has not been seen in *D. melanogaster, T. castaneum*, *C. capitata* or *An. stephensi* and is therefore of fundamental interest in understanding the dynamics of transposons [26].

While integrated *piggyBac* elements in the genome of *D. melanogaster* remobilize frequently enough for them to be valuable genomics tools in this species, *piggyBac* remobilization activity is highly variable among integrated elements and dependent, in part, upon the elements’ positions within the genome [27]. Esnault et al. (2011) saw a range of somatic and germ-line remobilization frequencies spanning two or three orders of magnitude for identical *piggyBac* elements and most of the observed variance could be attributed to the positions of the elements within the genome. They also showed, by moving elements with variable amounts of flanking chromosomal DNA to new genomic locations and measuring again the elements’ mobility, that less than a kilobase of flanking chromosomal DNA was responsible for determining the remobilization activity of an element [27]. Elements could be moved to different locations within the genome and retain their original remobilzation activities as long as approximately one kilobase of the original integration sites were included. Thus, there are intrinsic properties of the local flanking DNA that greatly impact *piggyBac* transposition activity either directly or indirectly.

Here we report that post-integration silencing of *piggyBac* mobility occurs in *Ae. aegypti* cells *in vitro*, that silencing occurs in the presence of functional transposase and that when silent elements are removed from the genome with approximately one kilobase of flanking chromosomal DNA and inserted into the genome of *D. melanogaster* they are fully active. Based on these data, it appears that the *piggyBac* elements analyzed in this study were silent only under some conditions, i.e. the genome of *D. melanogaster* provides DNA sequence contexts conducive to transposition whereas *Ae. aegypti* does not. The local neighborhood effects reported by Esnault et al (2011) appear to be due indirectly to intrinsic properties of local chromosomal DNA and the results reported here suggest that these effects may be species-specific. Alternatively, transpositional silencing of *piggyBac* may be due to larger scale features of the genome, epigenetic factors or systemic regulatory factors.

## Materials and Methods

### Plasmids

#### pBac:Act5cHyg:ie1EGFP

**Figure 1:**
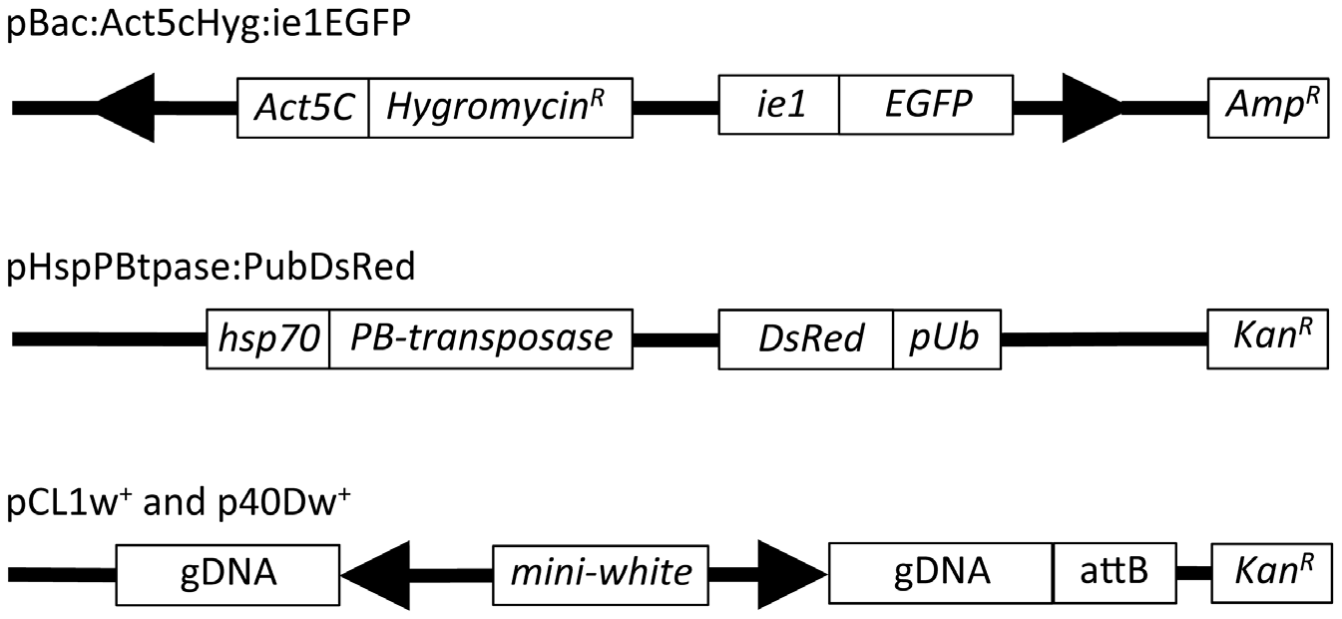
Plasmids used in this study. All plasmids are described in Material and Methods. Large arrowheads represent the terminal sequences of *piggyBac*. Act5C, promoter from *D. melanogaster* gene *Actin5C; Hygromycin^R^,* coding region for bacterial gene *hygromycin B phosphotransferase*; ie1, promoter from the baculovirus gene *immediate early 1*; Amp^R^, bacterial gene *beta-lactamase*; hsp70, promoter from *D. melanogaster* gene *hsp70*; PB-transposase, coding region for *piggyBac* transposase; DsRed, coding region for *Discosoma sp*. gene *red fluorescent protein*; pUb, promoter from *D. melanogaster* gene *pUbi-p63e*; Kan^R^, bacterial gene *Neomycin phosphotransferase II*; gDNA, refers to *Aedes aegypti* genomic DNA flanking the 5′ and 3 ends of *piggyBac* elements integrated in the genome of cell line AagPB8 (in pCL1w+) and in transgenic line 40D (in p40Dw+; [25]); mini-white, the *D. melanogaster* gene *w^+mW.hs^;* attB, the bacterial attachment site for phage *ΦC31.*

This 12 kb plasmid contains a functional *piggyBac* gene vector containing a *hygromycin*-*resistance* gene and the gene for the *Enhanced Green Fluorescent Protein* (*EGFP*) (Figure 1). It was constructed by subcloning a 4.5 kb fragment, *DmAct5c:hph:SV40*, containing the *D. melanogaster Actin5C* promoter regulating the expression of a *hygromycin phosphotransferase* gene and terminated by the 3′ UTR of SV40 from *pHermes:hph:tk* [28] into the *SacII* site of p*K[BIGα]* [29]. p*K[BIGα]* is a *piggyBac* vector with the *EGFP* gene under the control of baculovirus *hr4-ie1* promoter.

#### pHspPBtpase:PubDsRed

This 9.1 kb plasmid contains the *piggyBac* transposase gene under the control of the *hsp70* promoter from *D. melanogaster* and the fluorescent protein gene, *DsRed*, under the promoter control of the promoter from the *D. melanogaster polyubiquitin* (*pUb*) gene (Figure 1). This plasmid was constructed by inserting a 3.1 kb fragment from *pXL-BacII-PubDsRed* [30] containing *polyubiquitin*:*DsRed* into the *Eco*RI site of *pBac/hsΔsst* which contains the *piggyBac* transposase gene under the control of *D. melanogaster hsp70* promoter [31].

#### pCL1w+,p40Dw+

These are plasmids that contain identical *piggyBac* elements consisting of approximately 1 kb and 0.6 kb of the 5′ and 3′ terminal sequences, respectively, flanking the *D. melanogaster mini-white* gene along with approximately 1 kb of genomic DNA from their original integration sites in the genome of *Ae. aegypti*. pCL1w^+^ contains the *piggyBac* element from integration site 1110A in the transgenic cell line AagPB8 (see below) and has 1 kb and 500 bp *Ae. aegypti* genomic DNA flanking the 5′ and 3′ ends of *piggyBac*, respectively (Figure 1). p40Dw^+^ contains the *piggyBac* element from the transgenic *Ae. aegypti* line 40D (see below) and has 1 kb *Ae. aegypti* genomic DNA flanking the 5′ and 3′ ends of *piggyBac* (Figure 1).

The *piggyBac* elements in the plasmids described above were amplified by PCR from *Ae. aegypti* genomic DNA using *piggyBac* element-specific primers and *Ae. aegypti* genomic DNA specific primers. The reaction conditions for the PCR were: 1X Phusion Buffer (Finnzymes, Woburn, Massachusetts, USA), 0.2 mM of each dNTP, 0.1 µM forward primer and 0.1 µM reverse primer, 1 Unit Phusion DNA polymerase (Finnzymes, Woburn, Massachusetts, USA) and 10 ng of genomic DNA. MJ Research Thermo cycler was used to amplify the DNA under the following cycling conditions: 3 min at 98°C; 5 cycles of 15 sec at 98°C, 30 sec at 62°C, 5 min at 72°C; 35 cycles of 15 sec at 98°C, 30 sec at 68°C, 5 min at 72°C; 10 min at 72°C. The PCR fragments containing the 5′ terminal sequences of *piggyBac* and flanking genomic DNA had *AscI* and *PacI* restriction sites within the *piggyBac*-specific primers. These PCR fragments were ligated into pCR-Blunt II TOPO vectors as per the recommendations of the manufacturer (Invitrogen, USA). The PCR fragments of the 3′ terminal sequences of *piggyBac* with genomic DNA had *PacI* and *AscI* restriction sites in the *piggyBac*-specific primers and genomic DNA-specific primers, respectively. Following digestion with *PacI* and *AscI* these fragments were ligated into the *PacI* and *AscI* sites of the plasmids containing the 5′ terminal sequences of *piggyBac*, resulting in the creation of plasmids with functional *piggyBac* elements flanked by varying length of *Ae. aegypti* genomic DNA. The Drosophila mini-*white* gene was amplified from a plasmid PB [21] with primers containing *Pac*I restriction sites, digested with *Pac*I and ligated into the *Pac*I sites of the previous cloning step. This resulted in plasmids with *piggyBac* vectors carrying the mini-*white* gene flanked by various lengths of *Ae. aegypti* genomic DNA with a unique *Asc*I site in the plasmid backbone. The φC31 attB site was amplified from the plasmid pUASTattB [32] using PCR under the following conditions: 1X ThermoPol buffer (New England Biolabs, USA), 0.4 mM of each dNTP, 0.1 µM attBF primer and 0.1 µM attBR primer, 1u *Taq* polymerase (New England Biolabs, USA) and 5 ng of pUASTattB. The cycling conditions for the PCR reactions were: 5 min at 95°C; 25 cycles of 30 sec at 95°C, 30 sec at 56°C, 30 sec at 72°C; 5 min at 72°C. The resulting 300 bp fragment with *Asc*I sites at both ends was ligated into a pCR 2.1-TOPO vector (Invitrogen). The 300 bp fragment containing the φC31 attB site was removed with *Asc*I and ligated into the *AscI* site of the plasmids containing *piggyBac*-mini-*white* elements flanked by genomic DNA.

### Insect Strains

*Drosophila melanogaster* lines were obtained from the Bloomington Drosophila Stock Center at Indiana University and maintained at 25°C on a standard cornmeal/yeast-based diet. The following lines were used in this study:

### *Canton-S:* Wild-type

#### w1118

A homozygous recessive spontaneous loss-of-function mutation in the *white* gene resulting in white eyes containing less than 1% of normal pigmentation levels.

#### w1118; CyO, P0…2/wgSp-1

Multiple copies of a *P*-element containing the *piggyBac* transposase open reading frame under the regulatory control of the strong constitutive promoter from the *D. melanogaster tubulin* gene on the *CyO* balancer chromosome.

#### w1118; CyO, P0…2/wgSp-1; TM6BTbSb

Same as above with a 3^rd^ chromosome balancer.

#### P{ry+t7.2 = hsp70-flp}1, y1 w*; M{3xP3-RFP.attP}ZH-86Fb; M{vas-int.B}ZH-102D

Contains a φC31 attP site on the right arm of chromosome 3 and the φC31intgrase gene under the regulatory control of the promoter from the vasa gene on chromosome 4 [32].

#### CL1w+

P{ry+t7.2 = hsp70-flp}1, y1 w*; M{3xP3-RFP.attP}ZH-86Fb; M{vas-int.B}ZH-102D with an integrated copy of the plasmid pCL1w^+^.

#### 40Dw+

P{ry+t7.2 = hsp70-flp}1, y1 w*; M{3xP3-RFP.attP}ZH-86Fb; M{vas-int.B}ZH-102D with an integrated copy of the plasmid p40Dw^+^.

#### PBacw+

P{ry+t7.2 = hsp70-flp}1, y1 w*; M{3xP3-RFP.attP}ZH-86Fb; M{vas-int.B}ZH-102D with an integrated copy of the plasmid PB{RB} [21], [27].

#### A.a.40D

The transgenic *Ae. aegypti* line, 40D, contains a single *piggyBac* element with the *cinnabar* gene from *D. melanogaster* inserted into the genome of the *kh^w^* strain [25]. *Ae. aegypti* were reared under standard laboratory conditions as described [33].

### *Ae. aegypti* Cell Lines

#### Aag-2

An embryonic cell line maintained in Eagle’s Minimal Essential Medium (MEM) supplemented with MEM Non-Essential Amino Acids Solution, MEM vitamin solution, L-glutamine, and the antibiotics penicillin and streptomycin and 5% fetal bovine serum all cell culture reagents from Gibco®, Life Technologies) at 28°C in a 5% CO_2_ atmosphere [34], [35]. Cells were sub-cultured weekly at a 1∶5 dilution.

#### AagPB8

**Figure 2:**
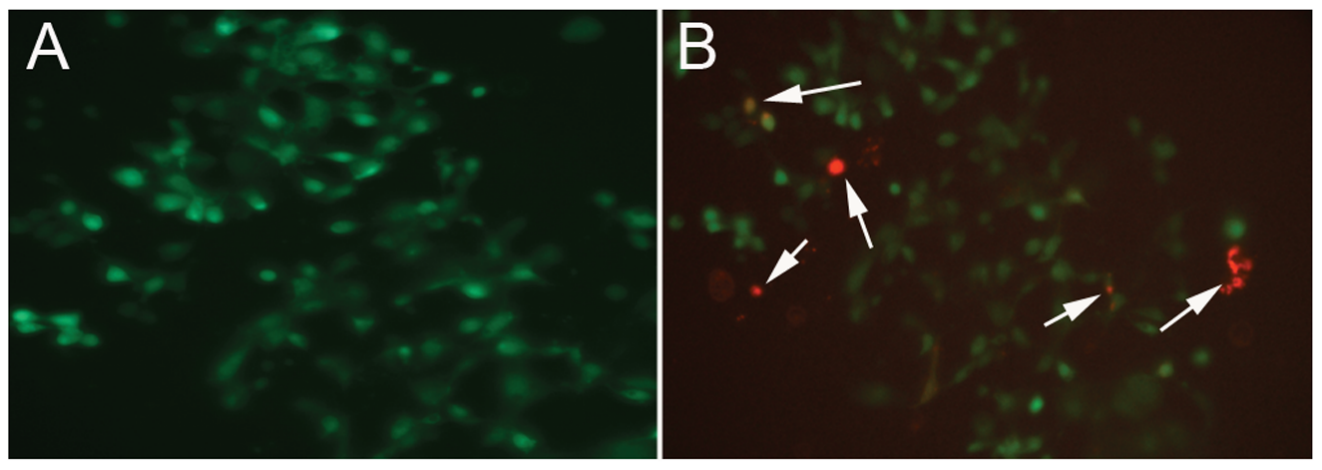
Cell line AagPB8. **A)** AagPB8 cells are Aag-2 cells containing 3 copies of an integrated *piggyBac* vector with a hygromycin-resistance gene under the regulatory control of the promoter from the cytoplasmic actin gene, *Act5C*, from *D. melanogaster* and EGFP under the regulatory control of the baculovirus *immediate early1* (*ie1*) promoter. Cells appear green because they were imaged under conditions allowing detection of fluorescence of EGFP. These cells were used for all experiments in this study. **B)** AagPB8 cells were transfected with *piggyBac*-expressing plasmids, pHspPBtpase:PubDsRed. The cells were heat shocked at 37°C for 1 hr 12 hrs post transfection and viewed using a florescence microscope to confirm the expression of EGFP (from the integrated *piggyBac* elements) and DsRed (from the transfected transposase-expressing plasmid or the negative control plasmid lacking transposase). Transfected cells expressing DsRed 12 hrs post heat shock are indicated with white arrows. The cells shown were sorted by FACS and used to assess transposition activity of *piggyBac* as described in the Material and Methods.

These are Aag-2 cells that contain 3 copies of an integrated *piggyBac* vector containing a hygromycin resistance gene under the regulatory control of the promoter from the cytoplasmic actin gene, *Act5C*, from *D. melanogaster* and EGFP under the regulatory control of the baculovirus *immediate early 1* (*ie1*) promoter (Figure 2). To create this cell line an equal volume of Lipofectin® Transfection Reagent (Invitrogen) and a mixture of p*Bac*:*Act5cHyg*:*ie1EGFP* and *pBac/hsΔsst* in Opti-MEM™ (Invitrogen, USA) was prepared such that the final concentration of each plasmid was 20 µg/ml and 10 µg/ml, respectively. One hundred microliters of this transfection mixture was added to the 900 µl of a suspension of Aag-2 cells in Eagle’s MEM (10^6^ cells/ml) lacking fetal calf serum, penicillin and streptomycin, and incubated in a 6-well cell-culture plate at 25°C, 5% CO_2_ for 12 hrs. After 12 hrs, the medium was replaced with Eagle’s MEM with 5% fetal calf serum and antibiotics penicillin and streptomycin and cells were heat-shocked at 37°C for 1 hr and then incubated for 2 weeks at 25°C, 5% CO_2_. The cells were split 1∶2 using Eagle’s MEM containing 5% fetal calf serum, penicillin, streptomycin and hygromycin B (50 µg/ml; Gibco®, Life Technologies). Hygromycin-resistant colonies appeared 4 weeks after selection with hygromycin B. Sixteen hygromycin-resistant monolayer colonies were individually picked and transferred to a well of a 96-well flat-bottom plate and allowed to grow. As cells overgrew the surface of the well they were transferred to progressively larger well and finally into a 25 ml cell-culture flask (Corning). Of the original 16 colonies, two (AagPB5 and AagPB8) were successfully amplified and established as a permanent cell line. AagPB8 was used for all experiments in this study.

#### *piggyBac* Excision Assay *in vitro*

**Figure 3:**
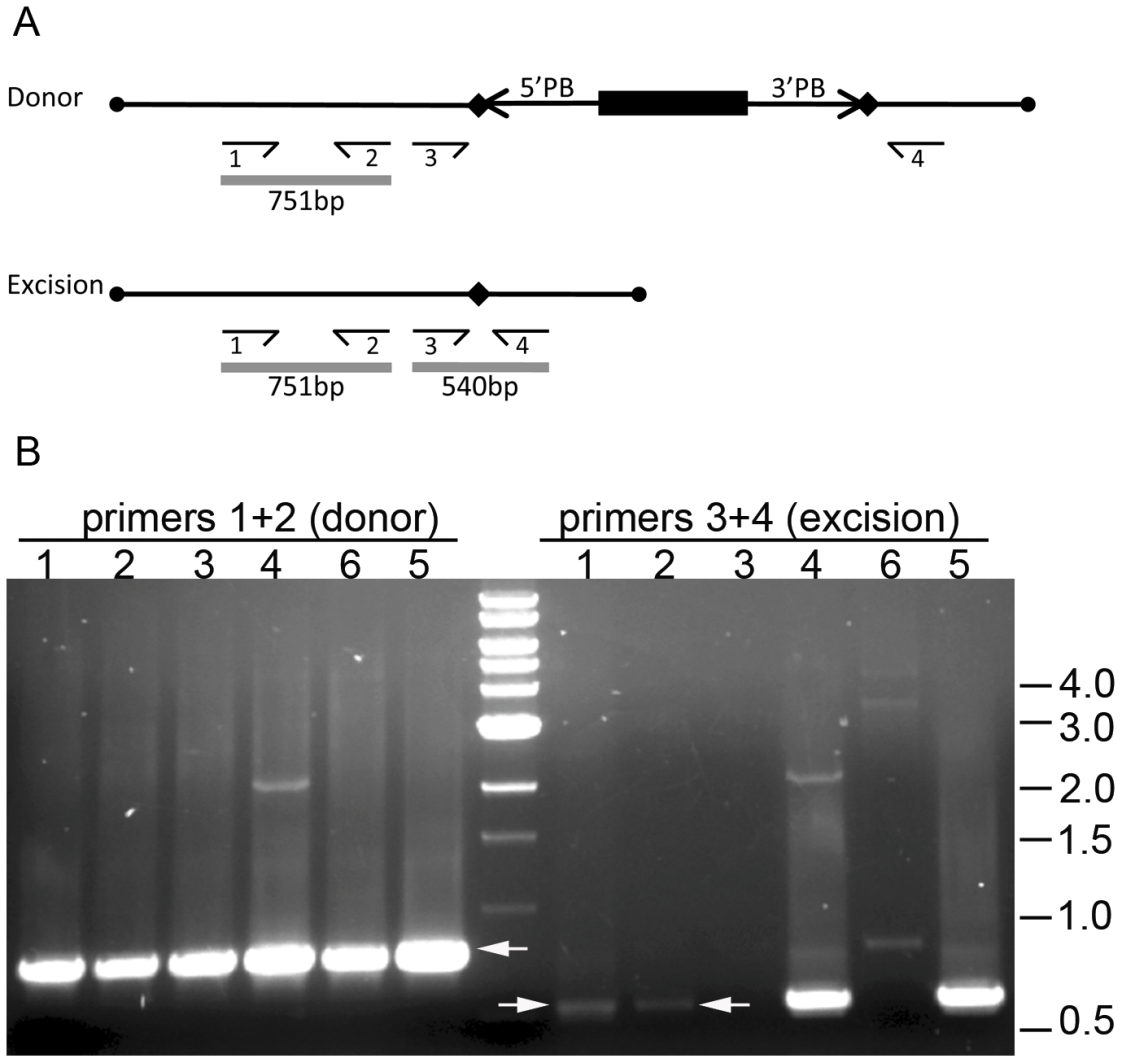
Plasmid-based *piggyBac* excision assay. A) Diagrammatic representation of the *piggyBac*-containing plasmid, *piggyBac* 3×P3EGFP used in the excision assay described in the Material and Methods (donor plasmid), and the same plasmid following precise excision of the *piggyBac* element (excision plasmid). The *piggyBac*-containing donor plasmid, *piggyBac* 3×P3EGFP, and *piggyBac* transposase expressing helper plasmid, pHspPBtpase:PubDsRed, were co-transfected into AagPB8 cells. Transfected cells were heat-shocked after 12 hrs and collected after 72 hrs. DNA was extracted and used as a template for PCR. Primers 1 and 2 (shown as labeled short half-arrows) were specific to the donor-plasmid backbone (494donorFWD, 494donorREV) and yield a 751 bp product (grey line) in the presence of the donor and excision plasmids. Primers 3 and 4 (494excisionFWD, 494excisionREV and shown as labeled short half-arrows) are specific to the plasmid DNA flanking the *piggyBac* element, however under the conditions of this experiment PCR products were only detected if donor plasmids missing the *piggyBac* element through excision were present, yielding a 540 bp PCR product (grey line). The 5′ and 3′ terminal *piggyBac* sequences are represented by arrows (5′PB, 3′PB). The duplicated TTAA target sequence into which *piggyBac* integrated is shown as a black diamond and the 3×P3EGFP transgene within the *piggyBac* element is shown as a black rectangle. The normally circular plasmids are represented as linear molecules. B) The PCR results from two *piggyBac* excision assays in AagPB8 cells. Lanes 1 and 2: from cells transfected with donor and *pHspPBtpase:PubDsRed* (2 independent transfections). Lane 3: from cells transfected with donor and control plasmids (pBluescript SKII+). Lane 4 and 5: positive controls for detecting excision events. The DNA used as a template in these reactions was a purified excision plasmid recovered from a previous excision assay (2 independent transfections). Lane 6: negative control for detecting excision events. DNA used as a template in this reaction came from cells transfected with donor plasmid only, without the transposase helper plasmid. Two PCR reactions were performed on each sample using primer combinations indicated above the lanes numbers. Primers 1+2 (same primers referred to in panel A) detected the presence of donor and excision plasmids and yielded a 751 bp reaction product (white arrow). Primers 3+4 (same primers referred to in panel A) yielded a 540 bp reaction product (white arrow) only when the *piggyBac* element in the donor plasmid had excised. Only the 540 and 751 bp bands are specific reaction products.

A plasmid-based *piggyBac* excision assay was performed as described previously but with minor modifications [36] and consisted of introducing a *piggyBac*-containing plasmid (*piggyBac* donor plasmid) and a plasmid that has a constitutively active copy of the *piggyBac* transposase gene (*piggyBac* helper plasmid) (Figure 3A). A mixture of these plasmids was introduced into cells and the plasmids subsequently recovered. Donor plasmids are analyzed by PCR for loss of the *piggyBac* element (excision). Specifically, one million AagPB8 cells were seeded into each well of a six-well cell culture plate 24 hr before transfection. One microgram each of the donor (pBac:3×P3 EGFP) [37] and helper plasmid (pHspPBtpase:pUbDsRed) were transfected into cells using of Lipofectin® Transfection Reagent (Invitrogen). After 12 hrs the cells were heat-shocked at 37°C for 1 hr. Cells were collected and plasmids were recovered from the cells at 72 hrs post-transfection using QIAprep Spin Miniprep Kit (Qiagen). Isolated plasmids were used as templates in two PCRs designed to detect the presence of intact donor plasmids and donor plasmids with from which *piggyBac* had excised. The presence of donor plasmids was confirmed by PCR using primers that would anneal to all donor plasmids and yield a fragment 751 bp in length (using primers 494donorFWD: TTTGCCGGATCAAGAGCTAC; 494donorREV: GCATTAATGAATCGGCCAAC) (Figure 3A). Donor plasmids that lost *piggyBac* by excision were detected by PCR using primers flanking the *piggyBac* integration site. In this PCR reaction intact donor plasmids yielded no products and plasmids lacking *piggyBac* yielded 540 bp fragments (using primers 494excisionFWD: CTTTATGCTTCCGGCTCGTA; 494excisionREV: ACGCGTATTAACGCAGAGTG) (Figure 3A). The PCR reactions included an initial denaturation step of 95°C for 2 min, followed by 40 cycles 95°C for 15 s, 54°C for 30 s and 72°C for 1 min with an elongation step at 72°C for 5 min. The PCR products were resolved and detected by agarose gel electrophoresis.

### *piggyBac* Transposition Assay *in vitro*

AagPB8 cells were seeded at the concentration of 10^6^ cells/well into six-well cell culture plates. One microgram of pHspPBtpase:PubDsRed was transfected into cells using Lipofectin® Transfection Reagent (Invitrogen). As a negative control, an equal number of cells were transfected with one microgram of pXLBacIIPubDsRed, which does not contain *piggyBac* transposase. After 12 hrs, the cells were heat shocked at 37°C for 1 hr and at 24 hrs after transfection the cells were viewed using a florescence microscope to confirm the expression of EGFP (from the integrated *piggyBac* elements) and DsRed (from the transfected transposase-expressing plasmid or the negative control plasmid lacking transposase). Transfection efficiency was approximately 10%. The cells were incubated at 25°C at 5% CO_2_ for an additional 72 hrs. The cells were collected, washed in Phosphate Buffered Saline (PBS; Gibco®, Life Technologies), pH 7.4 and resuspended in PBS. Cells expressing EGFP and DsRed were sorted using a FACSAria™ cell sorter (BD bioscience).

One thousand cells and 2×10^4^ cells were collected for whole genome amplification and genomic DNA isolation, respectively. Whole genome amplifications were performed on cell lysates using the Illustra GenomiPhi V2 DNA Amplification Kit according to the manufacturers recommendations (GE Healthcare). Genomic DNA was isolated using a DNeasy kit (Qiagen) after growing the initial 2×10^4^ cell for a week in Eagle’s MEM. Transposable element display was performed (see below) using amplified genomic DNA and genomic DNA as templates to test for the presence of *piggyBac* elements integrated in new locations within some of the recovered DNA.

### Transposable Element Display

This PCR-based integration site analysis method was performed as described by Sethuraman et al. (2007) with few modifications [25]. This genotyping method determines the number and location of *piggyBac* transposable elements within a genome based on the pattern of PCR fragments. Genomic DNA (25 ng) was digested with *Msp*I (New England Biolabs) according to the manufacturers recommendations. Sixty pmoles of MspI adapters (consisting of a dimer of oligonucleotides MspIa 5′GAC GAT GAG TCC TGA G 3′ and MspIb 5′CGC TCA GGA CTCAT 3′) were ligated to digested genomic DNA in the presence of *Msp*I overnight after which the reaction was diluted 4X with 0.1X TrisEDTA buffer (1 mM Tris HCL, pH 7.5, 0.1 mM EDTA). PCR (Pre-selective PCR) was performed using 5 µl of the diluted restriction/ligation reaction as template with primers MspIa and piggyL1 (5′TAT GAG TTA AAT CTT AAA AGT CAC G 3′) to display fragments containing the left inverted terminal repeat (ITR). The PCR reaction conditions were a denaturation step of at 95°C for 3 min followed by 25 cycles of 15 s at 95°C, 30 s at 54°C and 1 min at 72°C with a 5 min final elongation at 72°C. The pre-selective PCR products were diluted 20 times with 0.1X TrisEDTA buffer and 5 µl was used as a template in a PCR (Selective PCR) using primers MspI and Cy5-labelled piggyL5 Cy5 (5′ AGC AAT ATT TCA AGA ATG CAT GC 3′) to display fragments specifically containing the left inverted repeat. The selective PCR reaction conditions included an initial denaturation step of 95°C for 3 min, followed by 5 cycles of touchdown PCR consisting of a denaturation step of 95°C for 15 s followed by annealing for 30 s at a 59°C and an extension step of 1 min at 72°C, with the annealing temperature being reduced by one degree on each of five successive cycles. This was followed by 25 cycles of 95°C for 15 s, 54°C for 30 s and 72°C for 1 min final elongation step at 72°C for 5 mins. The selective PCR products were resolved on a 6% polyacrylamide gel under denaturing condition (6M urea). The gel was dried on 3 MM paper and viewed on a Storm 860 optical scanner (Molecular Dynamics) using the excitation wavelength of 635 nm. DNA bands of interest were excised from the gel, re-amplified using selective PCR protocol and unlabeled primers, purified using QIAquick Gel Extraction Kit (Qiagen) and sequenced.

### Creating Transgenic Drosophila Melanogaster

Two transgenic lines of *D. melanogaster* were created essentially as described by Esnault et al. (2011) [27]. Preblastoderm embryos of P{ry+t7.2 = hsp70-flp}1, y1 w*; M{3×P3-RFP.attP}ZH-86 Fb; M{vas-int.B}ZH-102D [32] were injected with *pCL1w^+^* and *p40Dw^+^* at 0.25 mg/ml and individual adults arising from these embryos were out-crossed to w^1118^ flies of the opposite sex. Red-eyed transgenic progeny of these crosses were used to establish homozygous lines (CL1w^+^ and 40Dw^+^).

### Genetic Analysis of piggyBac Activity

#### Somatic mosaicism assay

Males from the lines CL1w^+^, 40Dw^+^ and PBacw^+^ were crossed en mass with virgin females from the line *w1118; CyO, P0…2/wgSp-1*, which express *piggyBac* transposase. Curly-winged, red-eyed progeny were examined for the presence of clones of ommatidia with no pigmentation (indicates *piggyBac* excision) or with different grades of pigmentation more or less than the parental line (arising from *piggyBac* transposition and different levels of mini-*white* gene expression as a result of “position effects” [38]). Activity was expressed as the proportion of curly-winged, red-eyed progeny with eye-color mosaicism.

#### Germ-line activity

Males from the lines CL1w^+^,40Dw^+^ and PBacw^+^ were crossed en mass with virgin females from the line *w1118; CyO, P0…2/wgSp-1; TM6BTbSb*. Curly-winged, red-eyed, Sb progeny were crossed to w*1118. w^+^, Sb* progeny were scored as germ-line transposition events while *w, Sb^+^* progeny were scored as germ-line excision events.

## Results and Discussion

### *Aedes aegypti* Cell Line with Integrated *piggyBac* Elements – AagPB8

The cell line Aag-2 was co-transfected with the plasmids pBac:Act5cHyg:ie1EGFP and pHspPBtpase:PubDsRed and cells resistant to hygromycin B were selected. Foci of resistant cells were collected and subcultured to established hygromycin-resistant, EGFP-expressing cell lines (Figure 2). Cell line AagPB8 was used for all experiments and contains two integrated copies of pBac:Act5cHyg:ie1EGFP resulting from canonical cut-and-paste transposition of *piggyBac* and a third element associated with the integration of a copy of the plasmid pBac:Act5cHyg:ie1EGFP (Table 1). These data show that pHspPBtpase:PubDsRed, used as a source of *piggyBac* transposase in these cell line transfection experiments produces functional *piggyBac* transposase and that Aag-2 cells support *piggyBac* transposition.

**Table 1:** Location of *piggyBac* integration sites in AegPB8.

Element	Integration site	Position*	Location
CL1	ACTATAAAGCTTGTTTCAGTTTAACAAACTGTTGTTCTACTACT	supercont1.1100:32414	intergenic
CL2	TTTGTAGTCTTTTAAAGCTTTTAAAGAAATAAAGAAGGATTCGC	supercont1.160:2043689	intron

* Position of underlined nucleotide shown based on *Aedes aegypti* genome version 66.1 (AegL1)

### Mobility of Plasmid-borne *piggyBac* Elements in AagPB8

To test the ability of cell line AagPB8 to support *piggyBac* movement, plasmid-based *piggyBac* excision assays were performed. The plasmids pBac3×P3EGFP (donor) and pHspPBtpase:PubDsRed (helper) were co-transfected into AagPB8 cells and subsequently recovered. Using PCR primers that permit the detection of all donor plasmids (priming sites located within the plasmid backbone) and donor plasmids that had lost *piggyBac* through excision (priming sites immediately flanking *piggyBac*), PCR reactions were performed using DNA recovered from transfected cells (Figure 3A). In the presence of *piggyBac* transposase-expressing helper plasmids, donor plasmids that had lost *piggyBac* through excision were detected (Figure 3B). Excision products were not detected when the transposase-expressing helper plasmids were absent from the initial transfection mixture (Figure 3B). These data show that the cell line AagPB8, with three integrated *piggyBac* elements can support *piggyBac* movement of *piggyBac* elements located on plasmids.

### Immobility of Integrated *piggyBac* Elements in AagPB8

**Figure 4:**
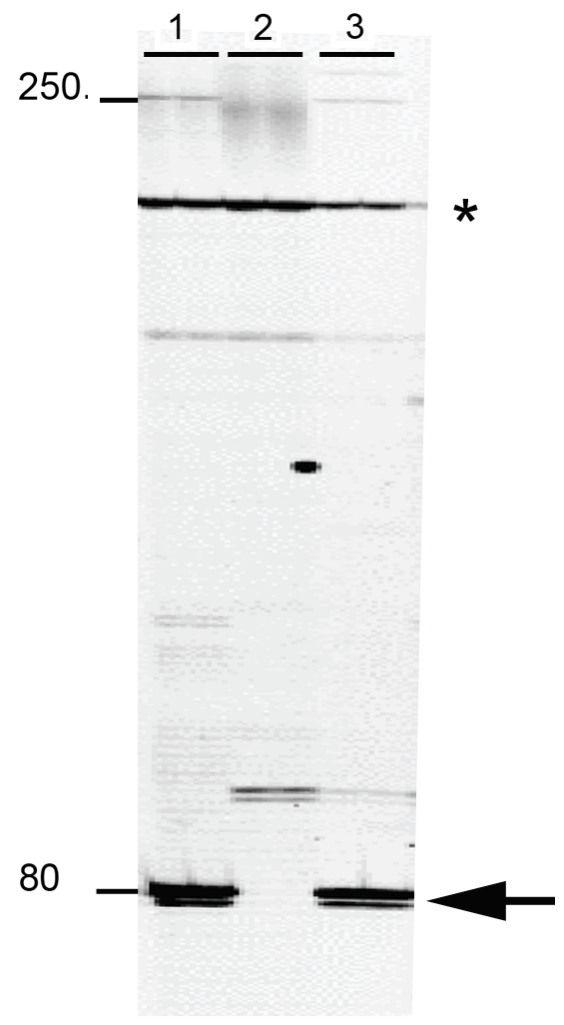
*piggyBac* transposable element display results using DNA isolated from cell line AagPB8. Lanes 1 are the results using DNA as a template isolated from cell line AagPB8 and shows evidence of the 5′ end of one of the two *piggyBac* elements that had integrated by canonical cut-and-paste transposition –80 bp band. The 5′ end of the second *piggyBac* element that integrated by canonical cut-and-paste transposition is not visible. This element can be detected when the 3′ ends of integrated *piggyBac* elements are visualized using transposable element display (not shown). The band at 250 bp is the *piggyBac* element associated with a copy of the integrated plasmid pBac:Act5cHyg:ie1EGFP. The sample was loaded into two adjacent lanes. Lanes 2 are the results using DNA as a template isolated from non-transgenic Aag-2 cells and this serves as a negative control for this assay since there are no *piggyBac* elements in *Ae. aegypti*. The sample was loaded into two adjacent lanes. Lanes 3 are the results using DNA as a template from AagPB8 cells 72 hours after being transfected with *piggyBac*-transposase-expressing pHspPBtpase:PubDsRed. The sample was loaded into two adjacent lanes. There was no evidence of *piggyBac* elements in other positions in the genome in Lane 3 as would be expected if *piggyBac* transposase mobilized the integrated *piggyBac* elements in AagPB8 cells. The asterisk indicated the position of a non-specific TE display band present in all samples. The positions of molecular weight markers 80 bp and 250 bp in length are shown.

Having confirmed that functional *piggyBac* transposase could be produced in cell line AagPB8 through transfection with pHspPBtpase:PubDsRed we measured the mobility of the chromosomally integrated *piggyBac* elements. Transposable element display is a sensitive method for detecting the presence of transposable elements in different positions within a genome. By starting with a genome in which the position of transposable elements of interest are known one can use transposable element display to detect the movement of transposable elements by comparing the position of the transposable elements at the beginning and end of an experiment. Transposable element display can be used not only to detect the movement of transposable elements in the germ line but also within somatic tissue. For example, Guimond et al (2003) were able to show extensive somatic activity of *Hermes* elements in transgenic Drosophila melanogaster [39]. We used a similar approach to test for *piggyBac* movement in the cell line AagPB8. In this case AagPB8 cells were transfected with the helper plasmid pHspPBtpase:PubDsRed and cells expressing *EGFP* and *DsRed* were collected and cultured (Figure 2). After cell sorting the cells expressing *EGFP* and *DsRed* were permitted to grow for 5–8 days after which time they were collected and their genomic DNA used in a transposable element display reaction. Based on TE display, no *piggyBac* elements in new locations were detected indicating that the chromosomally integrated *piggyBac* elements in AagPB8 were transpositionally silent (Figure 4). Therefore, integrated *piggyBac* elements in AagPB8 behave much differently in the presence of functional *piggyBac* transposase than do *piggyBac* elements in the same cells present on plasmids. Chromosomally integrated elements are unresponsive to functional transposase while plasmid-borne elements are mobile. These observations are consistent with those of Sethuraman et al (2007) who reported the transpositional silencing of integrated *piggyBac* elements in transgenic *Aedes aegypti* [25].

Two methods were used to assess *piggyBac* mobility, one based on movement of elements from plasmids and the other on the movement of element within genomes, and this raises the question as to whether differences in the sensitivity of these assays account for the differences in the observations reported. The plasmid assay gains it sensitivity from the large number of plasmids that can be introduced into cells. The intra-genomic mobility assay in combination with transposable element display gains its sensitivity from the ability to culture cells after introducing a source of transposase and amplifying the number of genomes with a *piggyBac* element in a new position. While both methods of assessing mobility would appear to be sensitive their relative sensitivities cannot be known and it should be considered a caveat when considering the conclusions reached here.

### Transpositionally Silent *piggyBac* Elements from AagPB8 can Transpose in *D. melanogaster*

Esnault et al (2011) found that local chromosomal DNA sequences immediately flanking integrated *piggyBac* elements in *D. melanogaster* account for almost all of the variance in element mobility observed among identical *piggyBac* elements integrated in different positions and exposed to a fixed source of *piggyBac* transposase [27]. Esnault et al (2011) could remove integrated *piggyBac* elements along with varying amounts of flanking DNA and re-integrate the elements and flanking DNA using site specific recombination into new positions within the genome of *D. melanogaster* [27]. As long as the elements retained approximately 1 kilobase of their original flanking genomic DNA they had the same remobilization activity as they did in their original position. Here we removed the *piggyBac* element CL1 from cell line AagPB8 and element 40D from Sethuraman et al’s (2007) transgenic *Aedes aegypti* line 40D, which was also shown to be completely stable in the presence of *piggyBac* transposase [25]. These elements were removed along with approximately 1 kb of flanking DNA and re-integrated into the same position within the genome of *D. melanogaster* using site specific recombination, as described by Esnault (2011) where their remobilization potential was measured in both the germ-line and soma as described [27].

The *piggyBac* elements CL1 from cell line AagPB8 and 40D from transgenic line 40D were highly active in both the soma and the germ line of transgenic *D. melanogaster*, in the presence of a constant source of transposase (Table 2). Ninety-six percent of *D. melanogaster* heterozygous for element CL1w+ and *piggyBac* transposase had eye-color mosaicism, indicating the frequent excision and transposition of the CL1w+ element. Similarly, 80% of *D. melanogaster* heterozygous for element 40Dw+ and *piggyBac* transposase had eye-color mosaicism. These rates of somatic *piggyBac* movement were higher than the control element PBbacw+ (78%) measured in this experiment and reported initially by Esnault et al (2011) at approximately 80%. *piggyBac* elements CL1w+ and 40Dw+ were also active in the germ-line of *D. melanogaster*. Germ line transposition and excision of CL1w+ was measured at 23% and 86%, respectively (Table 2). Transposition and excision of element 40Dw+ was observed in 4% and 16% of the germ-lines tested, while the control element, PBacw+, transposed and excised in 5% and 54% of the germ lines tested, respectively.

**Table 2:** Mobility of *piggyBac* elements with original *Aedes aegypti* flanking genomic DNA in *Drosophila melanogaster.*

		PBbacw^+a^	40Dw^+b^	CL1w^+c^
**Somatic**	**Mosaics** d	0.78 (0.04)	0.80 (0.02)	0.96 (0.06)
	**Crosses**	3	3	3
	**Progeny/Cross** e	133 (21)	134 (22)	94 (25)
**Germ-line**	**Germ-lines**	48	73	54
	**Excisions** f	26 (54.2)	12 (16.4)	49 (86.0)
	**Transpositions** g	9 (5.3)	3 (4.1)	13 (22.8)

a *piggyBac* from pPBac3×P3 EGFP with 1 kb of plasmid DNA flanking each terminal inverted repeat.

b *piggyBac* from transgenic *Aedes aegypti* line 40D with 1 kb of flanking genomic DNA.

c *piggyBac* element CL1 from *Aedes aegypti* cell-line AagPB8 with 1 kb of flanking genomic DNA.

d mean frequency of eye-mosaicism among progeny (standard deviation).

e mean number of progeny scored per cross (standard deviation).

f number of germ-lines yielding at least one excision event (percent).

g number of germ-lines yielding at least on transposition event (percent).

The *piggyBac* element CL1 in cell line AagPB8, and the *piggyBac* element in transgenic *Aedes aegypti* line 40D are both fully functional elements in chromosomal DNA contexts highly favorable for *piggyBac* excision and transposition, at least in *D. melanogaster*. The results reported here indicate that transpositional silencing of *piggyBac* elements upon integration into the genome of *Aedes aegypti* is a consequence of an unknown feature of the target site or chromosome, since we show here that 1) the integrated elements are structurally intact by demonstrating their mobility in *D. melanogaster*, 2) they are flanked by chromosomal DNA sequences that are conducive to element movement, 3) plasmid borne elements are active in the same cellular environment, and 4) functional transposase was provided in these experiments.

The basis for transpositional silencing of *piggyBac* is unknown and the experiments reported here, while not identifying the causal factors associated with silencing have suggested the involvement of higher scale genetic or epigenetic factors. These factors seem to affect the movement of any foreign transposable element introduced into the genome of *Ae. aegypti* and while perhaps reflecting an adaptive response to large transposable element movements earlier in the evolutionary history of the species, it currently impedes the development of powerful transposon-based functional genomics technologies.
